# Natural Polyamine Spermidine Inhibits the In Vitro Oxidation of LDL

**DOI:** 10.3390/molecules30040955

**Published:** 2025-02-19

**Authors:** Christine Rossmann, Azra Darko, Gerd Kager, Gerhard Ledinski, Willibald Wonisch, Thomas Wagner, Seth Hallström, Gilbert Reibnegger, Margret Paar, Gerhard Cvirn

**Affiliations:** 1 Division of Medicinal Chemistry, Otto Loewi Research Center, Medical University of Graz, 8010 Graz, Austria; christine.rossmann@medunigraz.at (C.R.); azra.darko@medunigraz.at (A.D.); gerd.kager@medunigraz.at (G.K.); gerhard.ledinski@medunigraz.at (G.L.); willibald.wonisch@medunigraz.at (W.W.); seth.hallstroem@medunigraz.at (S.H.); gilbert.reibnegger@medunigraz.at (G.R.); margret.paar@medunigraz.at (M.P.); 2 Department of Blood Group Serology and Transfusion Medicine, Medical University of Graz, 8010 Graz, Austria; thomas.wagner@medunigraz.at; 3 Division of Biomedical Research and Translational Medicine, Medical University of Vienna, 1090 Vienna, Austria

**Keywords:** antioxidants, atherogenesis, copper ions, endothelial damage, lipid oxidation

## Abstract

Spermidine is a natural autophagy-inducer and anti-aging compound. Herein, we investigated a potential autophagy-independent mechanism of spermidine, namely its capability to directly impede LDL oxidation, an early step in atherogenesis. In our in vitro-model, LDL oxidation was induced by the addition of CuCl_2_ in the presence of increasing concentrations of spermidine, and the degree of oxidation of the lipid, as well as of the protein part of LDL, was measured. We found that spermidine concentration-dependently inhibited the production of lipid hydroperoxides, malondialdehyde, and oxidation-specific immune epitopes in the LDL particle, associated with decreased relative electrophoretic mobilities, respectively. For example, the LPO content was significantly lower when LDL was oxidized in the presence of 500 µg/mL spermidine (26.9 ± 1.6 nmol/mg LDL) than in the absence of spermidine (180.6 ± 7.7 nmol/mg LDL, *p* < 0.0001). When oxLDL was obtained under increasing spermidine concentrations, its cytotoxicity in EA.hy926 cells concentration-dependently decreased. Quantum chemical calculations show that the reaction between spermidine and hydroxyl radicals is exergonic. We conclude that spermidine is a direct inhibitor of LDL oxidation due to its capability to scavenge hydroxyl radicals. Thus, spermidine supplementation might be a suitable tool to impede atherogenesis and associated (cardio)vascular diseases. Further prospective clinical studies are needed to evaluate the potential atheroprotective/health-promoting effects of spermidine-rich diets.

## 1. Introduction

Spermidine prolongs the life span in an autophagy-dependent manner, and, thereby, counteracts age-associated pathologies such as cardiovascular disease, neurodegeneration, and cancer [1]. Spermidine’s primary molecular mechanism appears to be boosting (macro)autophagy, a process by which cells eliminate dysfunctional or superfluous elements such as damaged proteins, cell membranes, and organelles [2]. Mechanistically, spermidine has been shown to mediate its effects via hypusination of the translation regulator eIF5A [2].

In addition, potentially autophagy-independent mechanisms have been proposed, including direct antioxidant and metabolic effects of spermidine. For example, enhancement of mitochondrial metabolic function and respiration, inhibition of neuronal nitric oxide synthase (nNOS), maintaining membrane potential, controlling intracellular pH and volume, anti-inflammatory, and anti-oxidative properties [3].

In the present study, we investigated one of these putative antioxidant properties of spermidine, namely whether it is able to prevent the oxidation of native low-density lipoprotein (nLDL), a fundamental step in the development of endothelial dysfunction and subsequent arteriosclerosis.

Dietary supplementation of spermidine has been shown to increase circulating spermidine levels, confirming its systemic bioavailability [4]. Thus, spermidine intake might be a suitable tool to attenuate, by virtue of inhibiting nLDL oxidation, the formation of arteriosclerosis with subsequent cardiovascular disease.

We aimed to examine the capability of spermidine to impede the oxidation of nLDL by using a well-established Cu^2+^ in vitro model [5]. After preincubating LDL with or without increasing spermidine concentrations, CuCl_2_ was added to oxidize it. Lipid hydroperoxide levels (LPO) and malondialdehyde (MDA) concentrations were measured to determine the extent of oxidation of the lipid part of the LDL particle. By measuring oxidation-specific immune epitopes and the corresponding relative electrophoretic mobility (REM), the oxidation of the protein part of the LDL particle was evaluated [6]. Furthermore, we used the MTT viability test to evaluate the cytotoxic effects of the oxLDLs obtained under increasing concentrations of spermidine in the vascular endothelial cell line EA.hy926 cells [7,8].

The chemical processes through which copper ions oxidize nLDL are not yet fully comprehended. Thus, it is somewhat challenging to clarify the exact reactions through which spermidine is thought to hinder Cu^2+^-mediated LDL oxidation. Copper ions are proposed to create initiating radicals through Fenton-type reactions or transition complexes with molecular oxygen, resulting in hydroxyl radicals (HO^•^), hydroperoxyl radicals (HOO^•^), and superoxide radical anions (O_2_^•−^) [9]. This study employed quantum chemical calculations to evaluate the reactivity of these reactive oxygen species (ROS) with spermidine, aiming to uncover potential mechanisms for the attenuation of LDL oxidation.

## 2. Results

### 2.1. Effect of Spermidine on the Oxidizability of the Lipid Part of the LDL Particle

**Figure 1:**
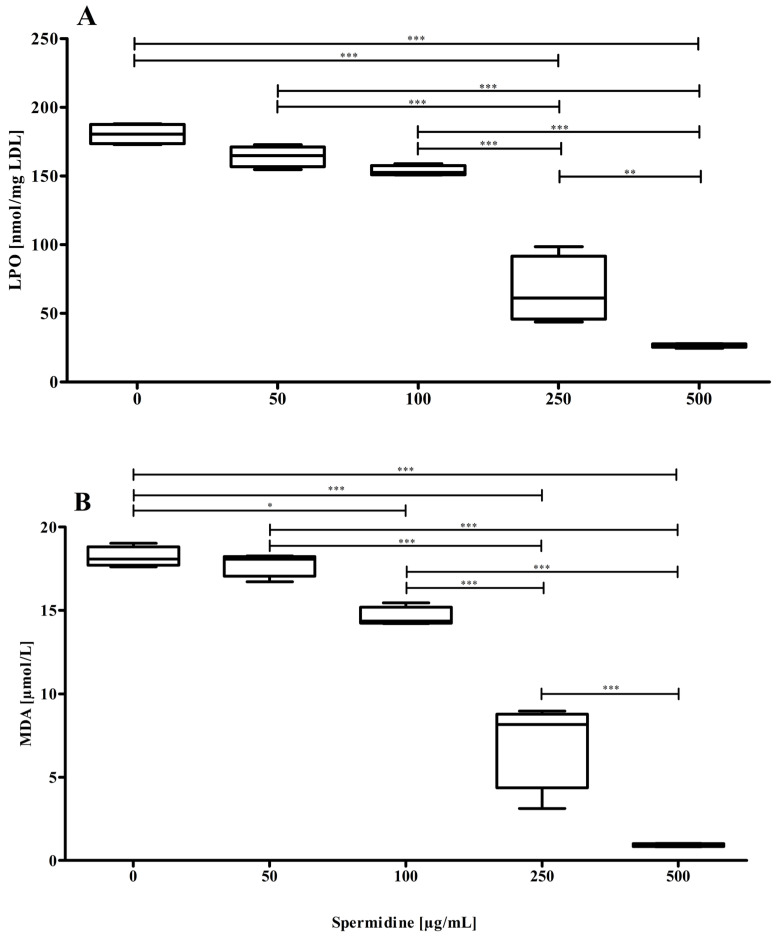
Cu^2+^-induced oxidation of the LDL particle’s lipid component. (**A**) Spermidine significantly (*p* < 0.0001, ANOVA with Bonferroni post-test) suppressed LPO formation; (**B**) Spermidine significantly (*p* < 0.0001, ANOVA with Bonferroni post-test) suppressed MDA formation after 4 h of incubation time. Data represent mean ± SD from four separate measurements; *… *p* ≤ 0.05, **… *p* ≤ 0.01, ***… *p* ≤ 0.001.

nLDL preparations were oxidized by addition of 10 µmol/L CuCl_2_ for up to 8 h in the absence or presence of spermidine (50, 100, 250, and 500 µg/mL). As demonstrated in our earlier study, the corresponding LPO content of LDL, which indicates the oxidative status of the lipid portion of the LDL particle, thereby increased time-dependently and reached a maximum between four and eight hours of incubation [10]. The oxidation of the lipid portion of the LDL particle caused by Cu^2+^ was inhibited by spermidine; the production of LPOs (Figure 1A) and MDAs (Figure 1B) decreased concentration-dependently with increasing spermidine concentrations.

### 2.2. Effect of Spermidine on the Oxidizability of the Protein Part of the LDL Particle

**Figure 2:**
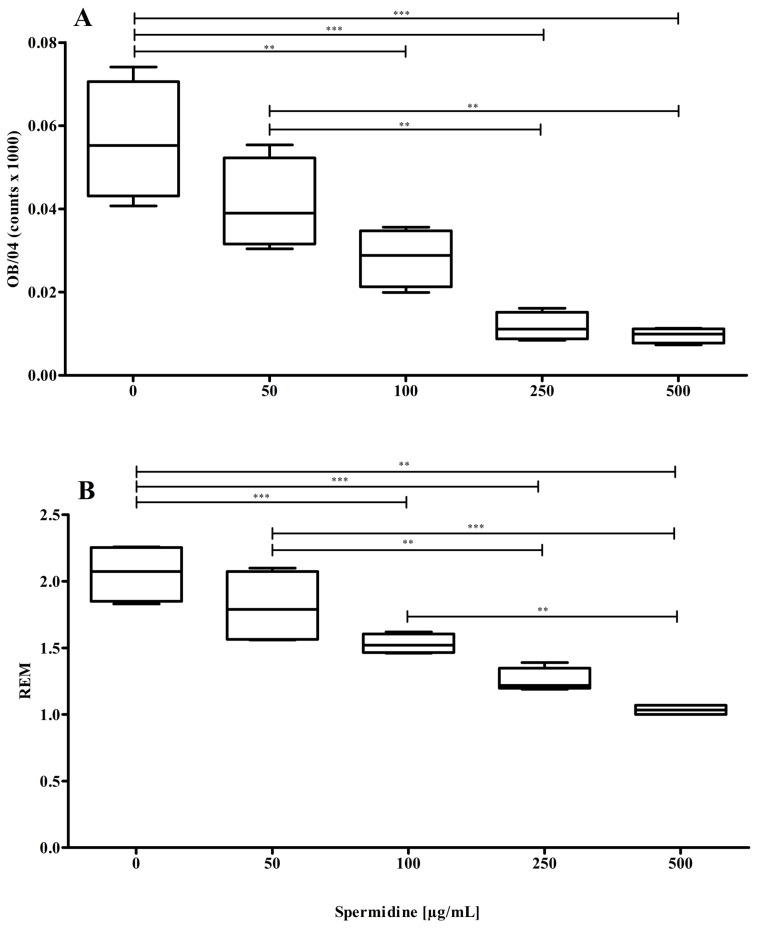
Cu^2+^—induced oxidation of the LDL particle’s protein component. (**A**) Spermidine significantly (*p* < 0.0001, ANOVA with Bonferroni post-test) suppressed the formation of oxidation-specific epitopes on the LDL particle; (**B**) spermidine significantly (*p* < 0.0001, ANOVA with Bonferroni post-test) reduced REM values after 4 h incubation time. Data represent mean ± SD from four separate measurements; **… *p* ≤ 0.01, ***… *p* ≤ 0.001.

As stated above, nLDL preparations were incubated with CuCl_2_ for up to eight hours with or without spermidine. As the spermidine concentration increased, the quantity of oxidation-specific epitopes decreased concentration-dependently (Figure 2A), suggesting that spermidine can inhibit the oxidation of the LDL particle’s protein component. As spermidine concentrations increased, REM values also decreased (Figure 2B), which is another indication that spermidine inhibits the oxidation of the LDL particle’s protein component.

### 2.3. Anti-Oxidative Action of Spermidine Compared to That of Alpha-Ketoglutarate (a-KG)

**Figure 3:**
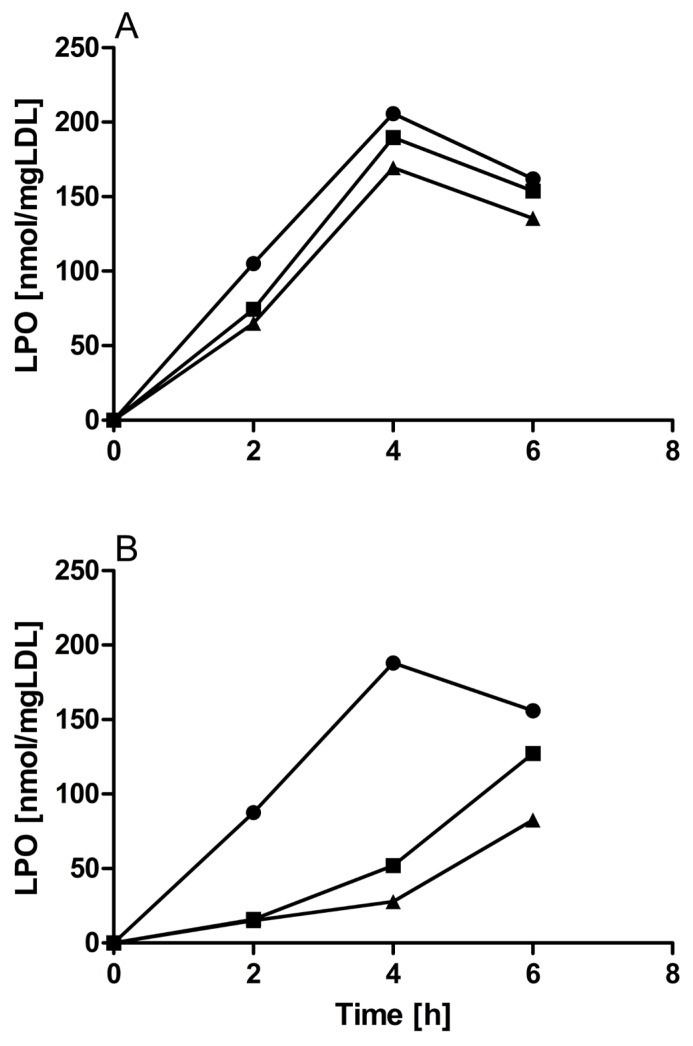
Suppression of LDL oxidation by spermidine vs. alpha-KG. (**A**) LPO formation in the absence (spheres) and in the presence of 250 µg/mL (squares) or 500 µg/mL (triangles) of alpha-KG; (**B**) LPO formation in the absence (spheres) and in the presence of 250 µg/mL (squares) or 500 µg/mL (triangles) of spermidine. Two typical experiments are shown.

Cu^2+^-initiated LPO formation in the LDL particle was concentration-dependently suppressed by a-KG, shown in Figure 3A. The same effect, but much more pronounced, was observed when spermidine was present during Cu^2+^-initiated oxidation of LDL (Figure 3B).

### 2.4. Cytotoxicity of oxLDL Formed Under Increasing Concentrations of Spermidine in EA.hy926 Cells

**Figure 4:**
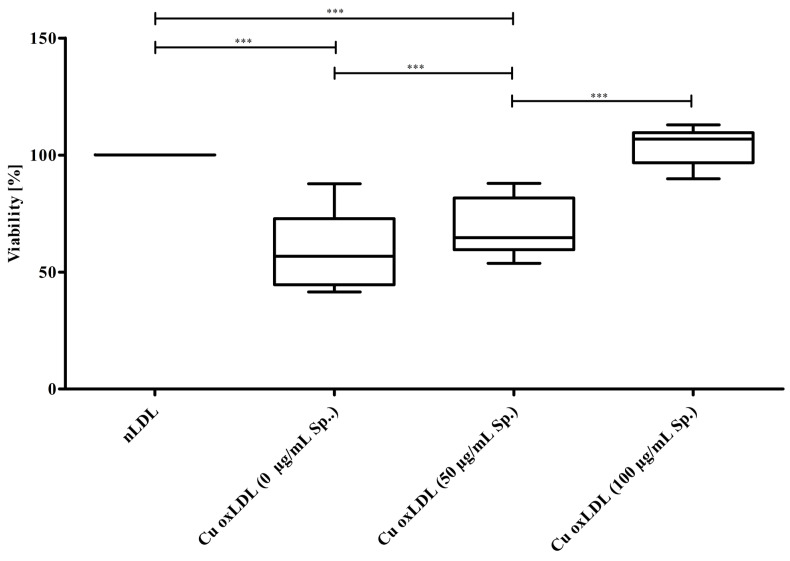
Spermidine during LDL oxidation reduces the cytotoxic effects of oxLDL. nLDL was preincubated with 0, 50, or 100 µg/mL spermidine and subsequently oxidized with CuCl_2_ until the LPO content without spermidine reached 100 nmol/mg LDL protein. The EAhy.926 cells were then incubated with the thus obtained oxLDLs (0.4 mg/mL). Cell viability concentration-dependently increased with oxLDLs formed under increasing levels of spermidine. Data represent mean ± SD from four separate measurements; ***… *p* ≤ 0.001. Cell viability values for incubation with nLDL was set to 100% for each experiment.

When oxLDL was produced during Cu^2+^-triggered oxidation of LDL in the presence of increasing spermidine concentrations, its cytotoxicity in EA.hy926 cells decreased concentration-dependently (Figure 4). Cell viability was significantly decreased in cells incubated with oxLDL, but it was recovered when oxLDLs produced with increasing spermidine concentrations were used.

### 2.5. Reactions of Spermidine with Hydroxyl Radical, Hydroperoxyl Radical, or Superoxide Radical Anion

As described above, Gibbs free energies for the abstraction of one hydrogen atom from spermidine by the hydroxyl radical (to produce water), by the hydroperoxyl radical (to produce hydrogen peroxide), or by the superoxide radical anion (to produce hydro-peroxide anion) were calculated and are displayed in Table 1.

**Figure 5:**
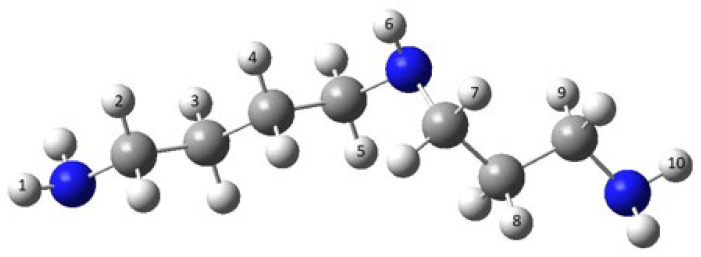
Chemical structure of spermidine. Large dark spheres: nitrogen; large light spheres: carbon; small spheres: hydrogen.

The abstraction of each of the three N-centered hydrogens (numbers 1, 6, and 10) as well as of seven methyl-hydrogens (numbers 2, 3, 4, 5, 7, 8, and 9) were studied, according to the scheme shown in Figure 5.

The only exergonic reactions were those between spermidine and HO^•^. The negative values for the corresponding Gibbs free energies in the polar (water) and apolar (benzene) milieus serve as evidence for this. The highest negative energy values are obtained by abstracting the N-centered hydrogen atom (number 6, Figure 5). Consequently, the best option for scavenging through spermidine is the HO^•^. Both HOO^•^ and O_2_^•−^ reactions with spermidine are endergonic (have positive values for their respective Gibbs free energies), which means they are unlikely to occur.

## 3. Discussion

In the present study, we show that the natural polyamine spermidine is capable of impeding the oxidation of nLDL, a crucial step in the development of endothelial dysfunction and atherogenesis [11,12,13].

By applying an in vitro model in which nLDL becomes oxidated by addition of Cu^2+^ ions [10], we show that spermidine concentration-dependently attenuated the oxidation of both the lipid and the protein moiety of the LDL particle. Consistent therewith, oxLDL was less cytotoxic in EAhy.926 cells when it was formed under increasing concentrations of spermidine.

Moreover, we show herein (by monitoring the inhibition of the formation of LPOs) that the antioxidant efficacy of spermidine is higher than that of the well-known antioxidant a-KG at the same concentrations.

Since the mechanisms by which Cu^2+^ initiates the oxidation of nLDL are not yet known completely, we can only speculate on how exactly spermidine limits the oxidation of nLDL. Previous studies suggest that three ROS in particular are at work: HO^•^, HOO^•^, or O_2_^•−^ [14,15]. Spermidine may presumably react with at least one of these radicals. Our calculations of reaction free energies identified the hydroxyl radical as the most likely candidate to be scavenged by spermidine. We, therefore, propose that the capability of spermidine to impede oxidation of nLDL is attributable, at least partially, to its capability to scavenge hydroxyl radicals. Our assumption is confirmed by the findings of previous studies showing that spermidine is capable of directly scavenging hydroxyl radicals [16,17]. Whereby, it is shown therein that spermidine is capable not only of scavenging hydroxyl radicals, but also of singlet oxygen.

Undoubtedly, the autophagy-promoting action of spermidine is the primary mechanism behind its health benefits [18,19]. In the present study, we point out that spermidine could have another health-promoting effect: direct attenuation of an early step in atherogenesis, namely the oxidation of nLDL. Atherosclerosis can easily lead to cardiovascular disease, which has reached epidemic proportions and is the worldwide leading cause of death [20].

It is generally believed that atherosclerosis represents a state of heightened oxidative stress characterized by lipid and protein oxidation in the vascular wall. The oxidative modification hypothesis of atherosclerosis states that nLDL oxidation is an early event in atherogenesis [13,21]. LDL lipids become oxidated while the LDL particle is traversing the subendothelial space of arteries. Consequently, apolipoprotein B100 lysine groups are modified so that the net-negative charge of the LDL particle increases. Thus, LDL becomes more susceptible to macrophage uptake via a number of scavenger receptor pathways producing cholesterol ester-laden foam cells [22]. This accumulation of foam cells form the nidus of a developing atherosclerotic lesion.

We assume that spermidine supplementation is a suitable tool to impede endothelial dysfunction/atherogenesis by virtue of its capability to impede nLDL oxidation. Spermidine has been suggested to be capable to enter (vascular) cells by means of the polyamine transporter system (PTS) [23]. Thus, spermidine apparently reaches the subendothelial space wherein it can unfold its atheroprotective actions. Toursarkissian et al. have shown that transmembrane polyamine transport occurs in aortic smooth muscle cells [24]. The antiatherogenic action of spermidine has already been shown in animal experiments: Spermidine reduced oxidative damage of endothelial cells in old mice [25] and alleviated the formation of atherosclerotic plagues in apoE-deficient mice fed a high-fat diet [16].

We assume that at least two interconnected reaction pathways presumably underlie spermidine’s antiatherogenic effects. Firstly, as has long been known, spermidine induces autophagy in cells involved in the development of arteriosclerosis, namely endothelial cells and vascular smooth muscle cells [26]. Secondly, as a conclusion that can be drawn from our results, spermidine causes direct suppression of the oxidation of nLDL in the subendothelial space of arteries, a highly pro-oxidative milieu [27], since it is capable to enter this space and to scavenge (at least) hydroxyl radicals.

It has been shown that oral supplementation of spermidine increases circulating spermidine levels, thereby confirming its systemic bioavailability [28]. Dietary spermidine could therefore be a suitable treatment in all clinical situations particularly associated with systemic inflammation and/or oxidative stress beyond atherosclerosis, e.g., rheumatoid arthritis (RA), diabetes mellitus (DM), obesity, or hypertension. For example, Nowak et al. have shown that oxLDL plasma levels are significantly elevated in RA patients [29].

Additionally, the physiological process of aging is known to induce a systemic proinflammatory state which has been shown to be associated with endothelial ROS formation through activation of nicotinamide adenine dinucleotide phosphate oxidase [29,30]. We propose that oral supplementation of spermidine, besides its autophagy inducing effects, might help to suppress the oxidation of nLDL, and, thus, the subsequent endothelial damage/atherogenesis in aging people. The systemic availability of spermidine comes from three equally significant sources: intestinal microbe production, external (oral) absorption with food, and cellular biosynthesis. Ingested spermidine has been shown to be quickly absorbed from the intestinal gut and distributed without degradation [31,32]. Supplementation of polyamine-producing probiotic bifidobacteria increases blood concentration of spermidine [33,34]. Thus, a polyamine-rich diet could overcome the age-associated decline of polyamines. Maintaining spermidine levels in aging may contribute to longevity.

The results presented herein might help to explain the clinical observation that dietary spermidine intake in humans inversely correlates with cardiovascular disease [20]. Whereby, the number of studies dealing with the health-promoting effect of spermidine in humans is still low to date. The results of our study may provide an incentive to conduct further prospective clinical studies, particularly in humans, to evaluate the potential atheroprotective/health-promoting effects of spermidine-rich diets. A strong argument for spermidine supplementation as a favorable treatment of diseases associated with inflammation/oxidative stress in future clinical trials is its low toxicity yet strong efficacy, even at moderate concentrations [1].

However, possible side effects have to be taken into account. Spermidine may act as a pro-oxidant rather than an antioxidant in the presence of free iron ions and H_2_O_2_ [35]. Moreover, possible gender differences must be taken into account in future clinical studies. It has been suggested that males and females might react differently to spermidine treatment [36,37].

## 4. Materials and Methods

### 4.1. Preparation of nLDL

The ethics committee of the Medical University of Graz approved this study, written informed consent was obtained from all participants (27-320 ex 14/15). Human LDL (1.020 to 1.063 g/mL) was obtained by sequential potassium bromide ultracentrifugation from the plasma of four young male normolipemic (Lp(a) < 5 mg/dL) individuals who were fasting for 12 to 14 h [5,7,38]. EDTA (1 g/L, Merck, Darmstadt, Germany), butylated hydroxytoluene (BHT, 20 µM, Sigma, St. Louis, MO, USA.), and Pefabloc (50 µM, Sigma Aldrich, Vienna, Austria) were used at every stage of lipoprotein preparation to stop lipid peroxidation and apolipoprotein B (apoB) cleavage by proteinases and potential contaminating bacteria. Before being used, the samples were sterile-filtered and kept in the dark at 4 °C. The protein content of LDL was measured using the Lowry method [39]. The CHOD-iodide test kit (Boehringer-Mannheim, Mannheim, Germany) was used to enzymatically determine the total cholesterol of the isolated LDL.

### 4.2. nLDL Oxidation Using Cu^2+^ Ions

nLDL (1.5 mg/mL) was preincubated with increasing concentrations of spermidine (0–500 µg/mL) for 30 min at 37 °C (pH = 7.4) in 0.01 mol/L phosphate buffer containing 0.154 mol/L NaCl. CuCl_2_ (10 µmol/L, final concentration) was then added to induce nLDL oxidation for up to eight hours. Concerning cell experiments, incubation with copper ions was stopped when LPO reached a threshold of 100 nmol/mg LDL in the absence of spermidine (controls). Subsequently, LDL was dialyzed over night at 4 °C against PBS.

### 4.3. Determination of Lipid Hydroperoxides (LPOs)

The LPO concentrations of the respective oxLDLs were determined with a spectrophotometric assay for lipid hydroperoxides in serum lipoproteins [7,38]. Accordingly, oxLDL solutions were mixed with a color reagent taken from the commercially available CHOD-PAP test kit and the absorbance was measured at 365 nm, as shown previously [7]. The concentration was calculated using the molar absorptivity of I_3_ ε = 2.46 × 10^4^ M^−1^cm^−1^. Calibration curves obtained with different peroxides such as H_2_O_2_, t-butyl hydroperoxide, and cumene hydroperoxide gave values for ε of 2.45 ± 0.04, 2.34 ± 0.26, and 1.26 ± 0.15 × 104 M^−1^cm^−1^, respectively. A stoichiometric relationship (slope = 1.02) was observed between the amount of organic peroxides assayed and the concentration of I_3_ produced.

### 4.4. Determination of MDA

MDA was determined according to a previously described HPLC method after derivatization with 2,4-dinitrophenylhydrazine (DNPH) [40]. Protein-bound MDA was hydrolyzed and deproteinized, as described previously [7]. For alkaline hydrolysis, 6 mol/L sodium hydroxide was used, and for deproteinization, 35% (*v*/*v*) perchloric acid was used. The supernatant was mixed with 12.5 µL DNPH solution and injected into the HPLC system (injection volume: 40 µL). The MDA standard was prepared on a 5 µm ODS hypersil column (150 mm × 4.6 mm) guarded by a 5 µm ODS hypersil column (10 mm × 4.6 mm; Uniguard holder; Thermo Electron Corporation, Cheshire, UK), as previously described [41]. The DNPH derivates (hydrazones) were isocratically separated on a 5 µm ODS hypersil column (150 mm × 4.6 mm) guarded by a 5 µm ODS hypersil column (10 mm × 4.6 mm; Uniguard holder) with a mobile phase consisting of a 0.2% (*v*/*v*) acetic acid solution (bidistilled water) containing 50% acetonitrile (*v*/*v*) at a flow rate of 0.8 mL/min at room temperature. The utilized HPLC consisted of an L-2200 autosampler, an L-2130 HTA pump, and an L-2450 diode array detector (all: VWR Hitachi; Vienna; Austria). Detector signals (absorbance at 310 nm) were recorded and EZchrom Elite software, version 3.3.2 SP2 (VWR, Vienna, Austria) was used for data acquisition and analysis.

### 4.5. Determination of Oxidation-Specific Immune Epitopes

As previously mentioned, a solid phase dissociation-enhanced lanthanide fluorescence immunoassay (DELFIA^®^) was utilized to track the development of oxidation-induced epitopes on apoB using monoclonal antibodies produced against modified apoB [6]. A monoclonal antibody called anti-ox-apoB (OB 04) was developed to combat copper-oxidized LDL and was shown to react selectively with lipoproteins that contained oxidized apoB [6]. The rabbit polyclonal antibody known as anti-apoB was acquired from Behring (Marburg, Germany). Both were used as detecting antibodies. Eu^3+^-labeled rabbit anti-mouse IgG (for OB/04) or Eu^3+^-labeled sheep anti-rabbit IgG (for anti-apoB) were used as reporting antibodies. As previously mentioned, the ratio of oxidatively modified LDL counts to nLDL counts was used to express the quantity of oxidation-specific epitopes on the LDL particle [7,42].

### 4.6. Determination of REM

The electrophoretic runs were conducted on a 1% agarose gel, and the lipoproteins were precipitated on the gel using the phosphotungstate-Mg^2+^ reagent. For 50 min, the electrophoresis was conducted at 100 V in 0.05 M barbital buffer. The ratio of the migration distances of oxLDL and nLDL was used to define REM.

### 4.7. Cell Culture

The American Type Culture Collection (ATCC) was the source of the EA.hy926 cells, which were kindly donated by Dr. C.J.S. Edgell of the University of North Carolina in Chapel Hill, NC, USA [43]. Primary human umbilical vein endothelial cells and a thioguanine-resistant clone of the human A549 cell line were fused to create EA.hy926 cells [43,44]. It has been demonstrated that EA.hy926 cells exhibit many characteristics of primary endothelial cells [44,45,46]. EA.hy926 cells were cultured, as described recently [7]. For up to eight hours, 0.4 mg/mL of nLDL and oxLDL—which is made by oxidizing nLDL with Cu^2+^ in the presence of increasing spermidine concentrations—were administered to the EA.hy926 cells.

### 4.8. Cell Viability (MTT Test)

The metabolic activity of EA.hy926 cells treated with Cu^2+^-oxidized LDL was assessed using the MTT (3-(4,5-dimethyl-2-thiazolyl)-2,5-diphenyl-2H-tetrazolium bromide) assay. Cells plated in 12 or 24 well plates were grown to confluence and then treated with the respective lipoproteins (in the absence or presence of spermidine) at concentrations of 0.3 mg/mL for the indicated time periods. MTT (1.2 mM in serum-free medium) was added to cells and incubated for 2 h at 37 °C under standard conditions. Cells were washed with PBS and cell lysis was performed with isopropanol/1 M HCl (25:1; *v*/*v*) on a microplate shaker at 1200 rpm for 15 min. Absorbance was measured at 570 nm on a Power Wave X Select microplate spectrophotometer (BioTek Germany, Bad Friedrichshall, Germany) and corrected for background absorption (650 nm).

### 4.9. Quantum Chemical Calculations

Becke’s three-parametric density exchange functional with the correlation function by Lee, Yang, and Parr (unrestricted for radical species, restricted for neutral species) was used in density functional theory (DFT) computations. To ascertain the thermal contributions to free energies, vibrational analyses and geometry optimizations were used in all calculations. The basis set for the calculations was 6–311+ G (2d,p). The SMD model was used to simulate either a polar (water) or nonpolar (benzene) environment in the calculations [47]. Delta G0-values, or changes in Gibbs free energies, were calculated for the reactions under standard conditions (298.15 K, 101.325 kPa). Electron densities, electrostatic potentials, and, in the case of open shell systems, spin density distributions were obtained for every molecular structure under study. The GAUSSIAN suite of quantum chemistry software (GaussView 6.0 and Gaussian G16 W, Gaussian Inc., Pittsburgh, PA, USA) was used for all quantum chemical computations and analysis of the findings.

### 4.10. Statistics

For statistical analysis, the GraphPad Prism Package (version 8.0) was utilized. The effects of increasing spermidine concentrations on markers of LDL oxidation and cell viability were statistically assessed using one-way ANOVA and Bonferroni post-tests. Statistical significance was set at *p* ≤ 0.05. *… *p* ≤ 0.05, **… *p* ≤ 0.01, ***… *p* ≤ 0.001.

## 5. Conclusions

Our study adds a further mechanism through which spermidine acts as an anti-atherogenic agent: its capability to impede oxidation of LDL through scavenging hydroxyl radicals. Spermidine could, therefore, be used as an antiatherogenic and geroprotective agent to promote healthy aging and extend life span.

## Figures and Tables

**Table 1:** Gibbs free energies of reaction. Negative values of Gibbs free energy denote exergonic reactions (abstraction of one hydrogen atom from spermidine). Positive values denote endergonic reactions.

Radical	Position of H-Abstraction	Gibbs Free Energy (kcal/mol)	
		Water	Benzene
	1	−25.01	−20.17
	2	−29.29	−28.69
Hydroxyl radical	3	−24.42	−22.20
	4	−25.25	−22.65
	5	−30.79	−29.24
	6	−32.12	−31.09
	7	−31.04	−29.99
	8	−24.49	−21.78
	9	−29.42	−28.85
	10	−25.58	−21.48
	1	08.48	13.30
Hydroperoxyl radical	2	04.19	04.79
	3	09.07	11.28
	4	08.24	10.83
	5	02.69	04.24
	6	01.36	02.38
	7	02.44	03.49
	8	09.00	11.70
	9	04.07	04.63
	10	07.90	12.00
	1	24.25	34.99
	2	19.96	26.48
Superoxide radical anion	3	24.84	32.96
	4	24.01	32.52
	5	18.46	25.92
	6	17.13	24.07
	7	18.21	25.18
	8	24.77	33.39
	9	19.84	26.32
	10	23.67	33.69

## Data Availability

The raw data supporting the conclusions of this article will be made available by the authors on request.
